# Impact evaluation of a brief online training module on physician use of the Maryland, USA, Prescription Drug Monitoring Program

**DOI:** 10.1371/journal.pone.0272217

**Published:** 2022-08-09

**Authors:** Oluwasanmi O. Adenaiye, Julia B. Zirpoli, Marissa Tan, Brendan F. Day, Olayiwola Bolaji, Clifford S. Mitchell, Marianne Cloeren

**Affiliations:** 1 Institute of Applied Environmental Health, School of Public Health, University of Maryland, College Park, Maryland, United States of America; 2 School of Medicine, University of Maryland, Baltimore, Maryland, United States of America; 3 Department of Epidemiology and Public Health, University of Maryland School of Medicine, Baltimore, Maryland, United States of America; 4 Internal Medicine Department, University of Maryland Capital Regional Health, Cheverly, Maryland, United States of America; 5 Environmental Health Bureau, Maryland Department of Health, Baltimore, Maryland, United States of America; 6 Division of Occupational and Environmental Medicine, Department of Medicine, University of Maryland School of Medicine, Baltimore, Maryland, United States of America; King Abdulaziz University Faculty of Medicine, SAUDI ARABIA

## Abstract

**Background:**

Prescription Drug Monitoring Programs (PDMPs) are electronic databases that track controlled substance prescriptions in a state. They are underused tools in preventing opioid abuse. Most PDMP education research measures changes in knowledge or confidence rather than behavior.

**Objective:**

To evaluate the impact of online case-based training on healthcare provider use of the Maryland (USA) PDMP.

**Methods:**

We used e-mail distribution lists to recruit providers to complete a brief educational module. Using a pre-training and post-training survey in the module, we measured self-reported PDMP use patterns and perceived PDMP value in specific clinical situations and compared pre- and post-training responses. Within the module, we presented three fictional pain cases and asked participants how they would manage each, both before, and then after presenting prescription drug history simulating a PDMP report. We measured changes in the fictional case treatment plans before and after seeing prescription history. Finally, we measured and compared how often each participant accessed the Maryland PDMP database before and after completing the educational module. We used multivariate logistic regression to measure the effect of the intervention on actual PDMP use frequency.

**Results:**

One hundred and fifty participants enrolled and completed the training module, and we successfully retrieved real-world PDMP use data of 137 of them. Participants’ decisions to prescribe opioids changed significantly after reviewing PDMP data in each of the fictional cases provided in the module. In the months following the training, the rate of PDMP use increased by a median of four use-cases per month among providers in practice for less than 20 years (*p* = 0.039) and two use-cases per month among infrequent opioid prescribers (*p =* 0.014).

**Conclusion:**

A brief online case-based educational intervention was associated with a significant increase in the rate of PDMP use among infrequent opioid prescribers and those in practice less than 20 years.

## Introduction

Opioid prescribing has been declining since 2012 in the United States and in Maryland [1], but opioid overprescribing continues to contribute to the wicked public health problem of opioid overdose deaths [2–5]. The state of Maryland had decreases in prescription opioid-related deaths from 2016 through 2018, but no change from 2018 to 2019 [6].

Many studies have shown an increased risk for long-term work disability in patients prescribed opioids in workers’ compensation (WC) injuries, with associated increased costs [7–10]. The Workers’ Compensation Research Institute (WCRI) reviewed data from 28 states on low back injuries that had at least seven days away from work between 2008 and 2013 and found three times as much temporary disability in workers prescribed opioids as in similarly injured workers who did not receive any opioid prescriptions [11]. WCRI found that 21% of WC claims in Maryland for lost time injuries occurring between October 1, 2015, and September 30, 2016, received an opioid prescription [12]. States implementing opioid prescription guidelines in WC have seen improved outcomes in work disability and other metrics [13, 14].

**Fig 1:**
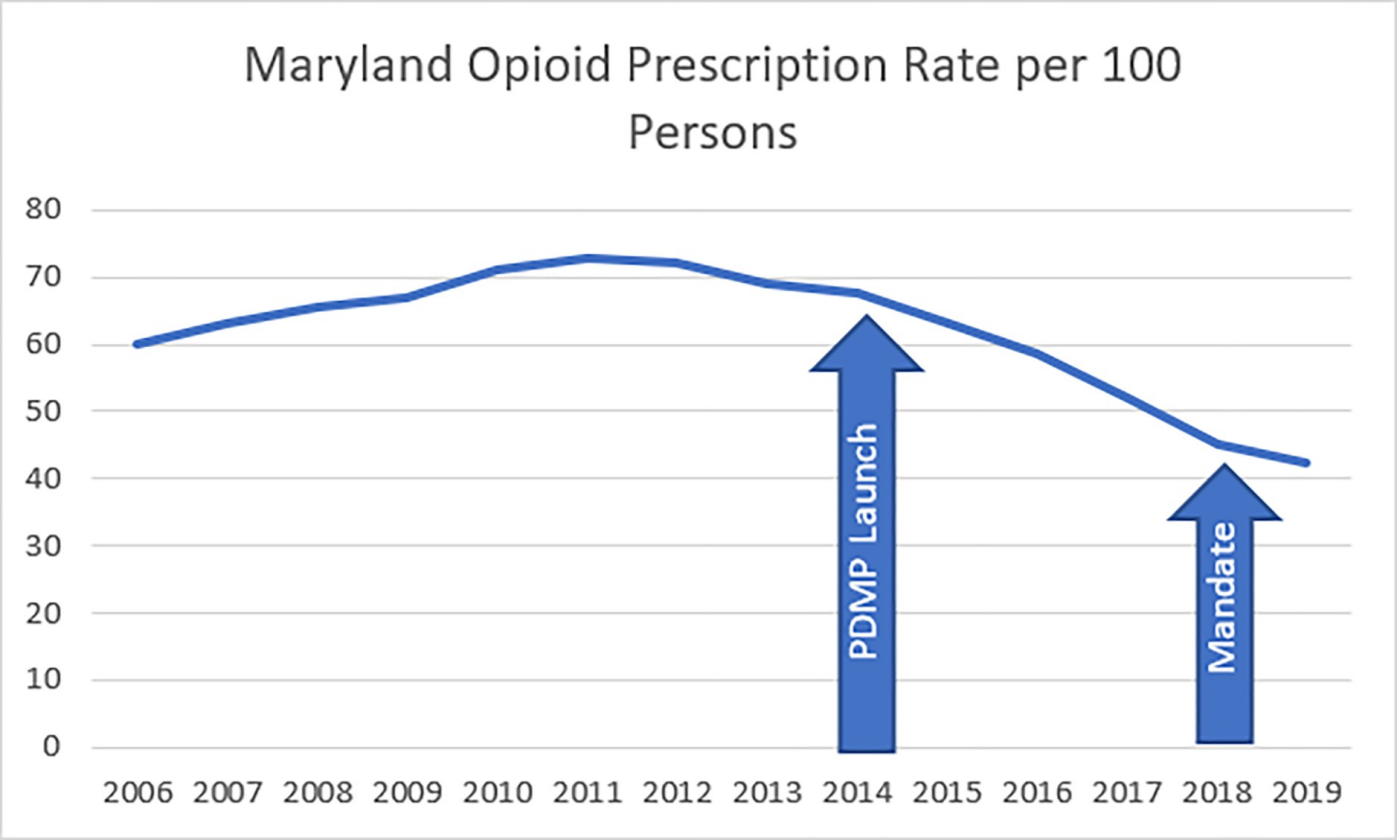
Maryland opioid prescription trends. Maryland opioid prescription rate in relation to PDMP milestones [17].

Prescription drug monitoring programs (PDMPs) are statewide electronic databases that track prescriptions for controlled substances. They are intended to improve safe prescribing of opioids by identifying patients who may be misusing opioids or other controlled prescription drugs and those at increased risk for opioid overdose [15]. Maryland established its PDMP in 2014 and requires all clinicians who may prescribe controlled drugs to register for access. In July 2018, Maryland mandated that providers check the PDMP before prescribing a controlled drug to a patient [16]. Maryland opioid prescription rates had already started declining before the PDMP was launched [17]. Fig 1 illustrates opioid prescription trends in Maryland in relation to the launch of the PDMP in 2014 and the mandate implementation in 2018.

Several studies of PDMPs have noted population-level benefits following PDMP implementation, including decreased opioid prescribing [18–20], opioid misuse [21], opioid-related deaths [20, 22], and patients seeing multiple prescribers for the same drug [20, 21]. However, others have failed to demonstrate a clear benefit [23–25] and even suggested potential unintended harm of increasing heroin use [23]. Studies have shown that providers feel PDMPs are beneficial [26, 27] but are often difficult to use [19, 27, 28]. Interventions to improve provider use of PDMPs have suggested some improved PDMP knowledge with educational interventions done face-to-face [29], through interactive webcasts [30], or online slide presentations [31, 32]. One recent study on the effectiveness of academic detailing, involving a one-to-one meeting between a trained pharmacist and primary care providers, demonstrated increased PDMP use in the 6-month follow-up period [33]. A recent systematic review on barriers to PDMP use found that the most reported system training barriers were lack of knowledge on how to use the PDMP, lack of access training, lack of training on how to interpret results and communicate results to patients and the lack of consistent guidelines on when to check the PDMP [34].

Research on the effectiveness of different physician education modalities, including online training, has often been limited to measuring increases in knowledge or confidence [35–37]. Assessing training impact on clinician behavior and patient outcomes is more challenging [38]. There is evidence to support the value of more active learning strategies, in which learners interact by making decisions, not just reading, viewing, or listening to content [39]. Simulations involving standardized patients are resource-intensive and logistically challenging for reaching clinical providers in practice. Researchers in medical education are hoping to replicate the success of simulated in-person training using more cost-effective and flexible online interactive training [40, 41].

We developed the current project to evaluate the impact of a brief, case-based module on Maryland prescribing providers’ medical decision making as well as PDMP attitudes, behaviors, and utilization. This module was based on a similar module published by one of the authors (MC) for the (now defunct) online clinician community of practice QuantiaMD in 2015. The module presents fictional case studies with medical decisions to be made before and after receiving PDMP data in each case, inviting learners to reflect on the impact of that information on their own medical decision making, rather than providing a “correct” answer, as does the design of most other online education. The previously published module is no longer available online, but it had a high level of provider engagement and expressed intent to change behavior (https://www.fiercepharma.com/sponsored/how-digital-content-helping-hcps-curb-opioid-epidemic.) The Maryland Department of Health was interested in testing a similar module, with a focus on WC care, in a way that would permit measurement of change in attitudes and behavior of participants. We hypothesized that this training module would increase participant recognition of the indications for using the PDMP and increase participant use of the PDMP in practice.

## Materials and methods

### Module development and launch

We developed the educational module and published it in Sharable Content Object Reference Model (SCORM) format, using the e-learning authoring tool iSpring Suite 9.0) [42]. We modified the module to include content about the Maryland PDMP and to use work-related condition scenarios in the cases. Maryland PDMP program managers reviewed the content about the PDMP for accuracy. University of Maryland School of Medicine faculty and residents beta-tested the module prior to launch.

The module included:

- A baseline questionnaire capturing minimal practice demographic information; self-reported WC services, opioid prescription frequency, and PDMP utilization; attitudes about the value of the PDMP in specific clinical situations; and self-reported PDMP use behavior in these clinical situations.
- Orientation to the Maryland PDMP.
- Three fictional cases featuring painful WC clinical situations, with clinical decisions (including opioid prescription) to be made before and after receiving PDMP data.
- A post-training questionnaire measuring attitudes about the value of the PDMP in specific clinical situations, and planned use of the PDMP in those situations.
- Optional provision of e-mail address if willing to complete a follow-up survey.

A link to the module and the survey questions are found in the Supplemental Materials.

### Approvals

We obtained approval for human subjects’ research, with a waiver of signed consent, from the Institutional Review Boards of the University of Maryland Baltimore (HM-HP-00076603-2) and the Maryland Department of Health (MDH/BHA 17–75). We obtained continuing medical accreditation for this module from MedChi, the Maryland Medical Society.

### Sample size calculations

Using G-power® [43], we estimated the needed sample size from results from a similar educational module administered to 16,861 clinicians in an online community of practice. The proportion of participants who said they would use the PDMP increased from 33% at baseline to 55% post-module. Hence, to detect a difference of δ = *p*1 – *p*0 = 0.22 (where *p*1 and *p*0 = proportions of subjects with an intention to use the PDMP post-training and pre-training respectively), with 80% power and 95% confidence level, we estimated we would need a sample of at least size n = 116 [44].

### Recruitment

Eligibility criteria were having a license to practice/prescribe in Maryland and having a Maryland Controlled Dangerous Substances (CDS) registration. We recruited Maryland health care providers via e-mail using:

- Blast messages from the Maryland State Medical Society to its member list
- Announcements to the University of Maryland Medical System physician list
- Messages to members of Maryland medical societies
- Notices to members of the Maryland Workers’ Compensation Education Association
- Outreach via contacts at local hospitals to send notice to their clinical staff.

An e-mail message directed potential participants to the project landing page, which described the study, including planned use of data and protections. By clicking on a link that directed a participant to register for the training module, a participant consented to use of their data collected within the module and to the research team collecting information on their use of the PDMP before and after taking the training. We launched the training module on iSpring Learn (https://www.ispringsolutions.com/support/learn), a subscription online learning management system (LMS), which supported registration, delivery of the module, bookmarking, tracking progress and completion, and generation of a certificate for Continuing Medical Education upon completion. Participants could complete the module, which took about 30 minutes to complete including the questionnaires, at their discretion, using mobile technology if they wished. We sent out reminders to participants who started but did not complete the training. Recruitment continued for 10 months and we compensated participants with continuing medical education credits.

### Data collection

**Fig 2:**
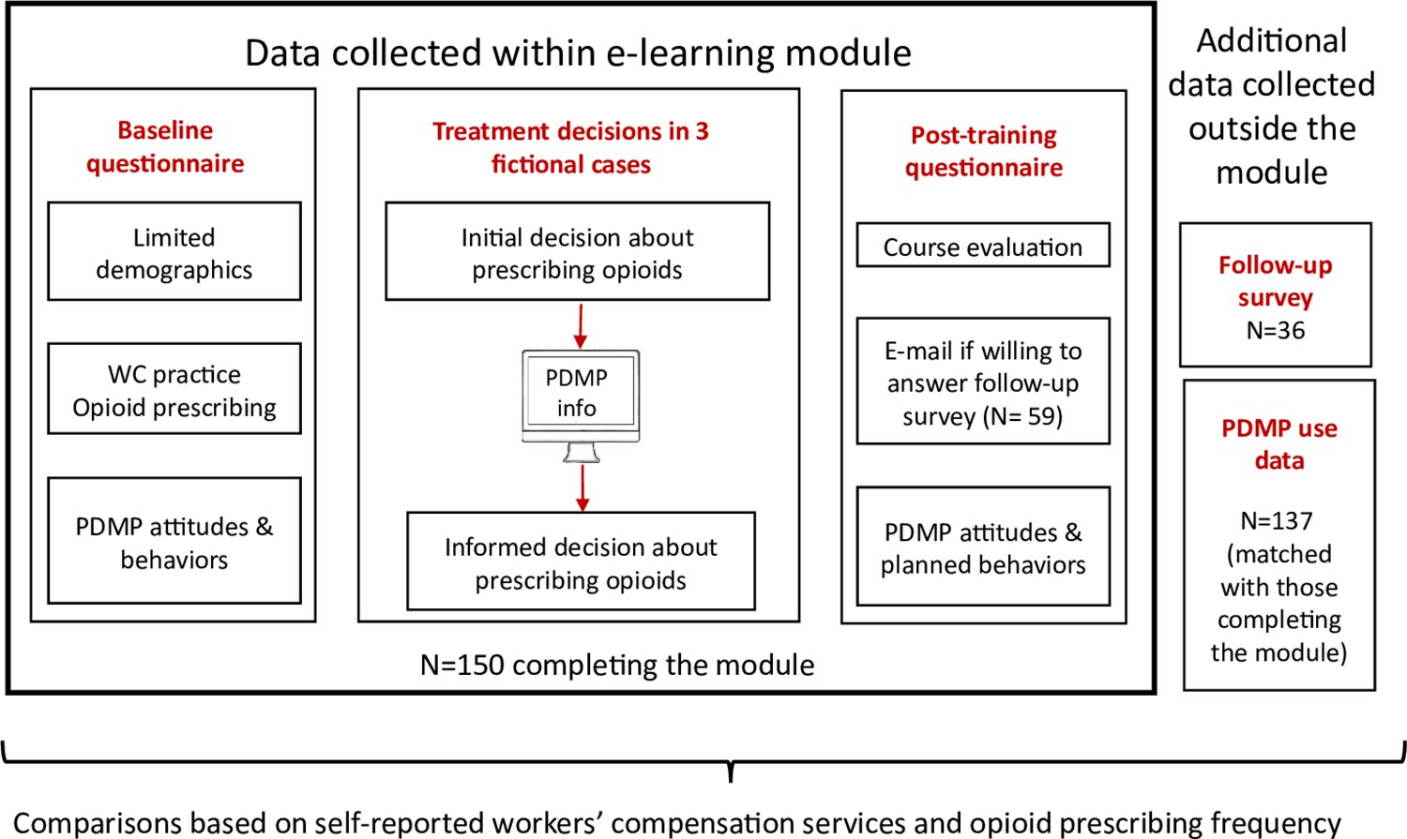
Data sources within and external to module.

The LMS collected all responses entered by participants in the module, including the baseline questionnaire, treatment decisions in the fictional cases, and the post-training survey. We began recruitment and data collection from the module in June 2018 and ended it in March 2019. We retrieved participants’ 6-month pre-training and up to 6-months post-training PDMP use data from the Maryland PDMP database of Chesapeake Regional Information System for our Patients (CRISP) using their CDS numbers. Other sources of data were publicly available registries of Maryland physicians and reports on registration for the Maryland PDMP [45] and responses to a post-training follow-up survey sent to a subset of participants who provided their e-mail addresses for this purpose. Fig 2 describes the collection of data used for analysis within and external to the module.

### Data processing and statistical methods

We categorized the participants completing the module based on the self-reported frequency of opioid prescribing and PDMP use as “frequent” (several times per day; several times per week; about once a week) or “infrequent” (a few times a month; about once a month; a few times per year or less), as well as on self-reported delivery of WC related services in the previous year. We used McNemar’s test to compare participants’ responses to the survey before and after the module and their treatment plan responses in the fictional cases.

We used chi-square test to compare demographic differences in PDMP registration status between participants and licensed Maryland physicians and to compare participants’ responses to demographic questions.

For the participants with PDMP use data, we tested the differences between the rate of PDMP use up to six months before and six months after taking the module using Wilcoxon signed-rank test. To control for the effect of the mandate requiring providers to check the PDMP when prescribing controlled substances, we excluded from the analyses participants who took the training before the mandate implementation date. We re-computed the analyses on a subgroup of participants who took the training at least six months following the mandate. We ran multi-model logistic regression analyses to determine the odds of observing a change in the rate of PDMP use (i.e., an increase of at least one-use case per month) following training. We used a backward elimination model selection method to select the most predictive model.

Statistical significance was assessed using two-tailed tests at a significance level of 0.05. We used R software version 3.6.3 (RStudio, Inc., Boston, MA, USA) for data management and both R and Statistical Analysis Software (SAS) version 9.3 (Cary, NC, USA) for statistical analyses [46].

## Results

**Fig 3:**
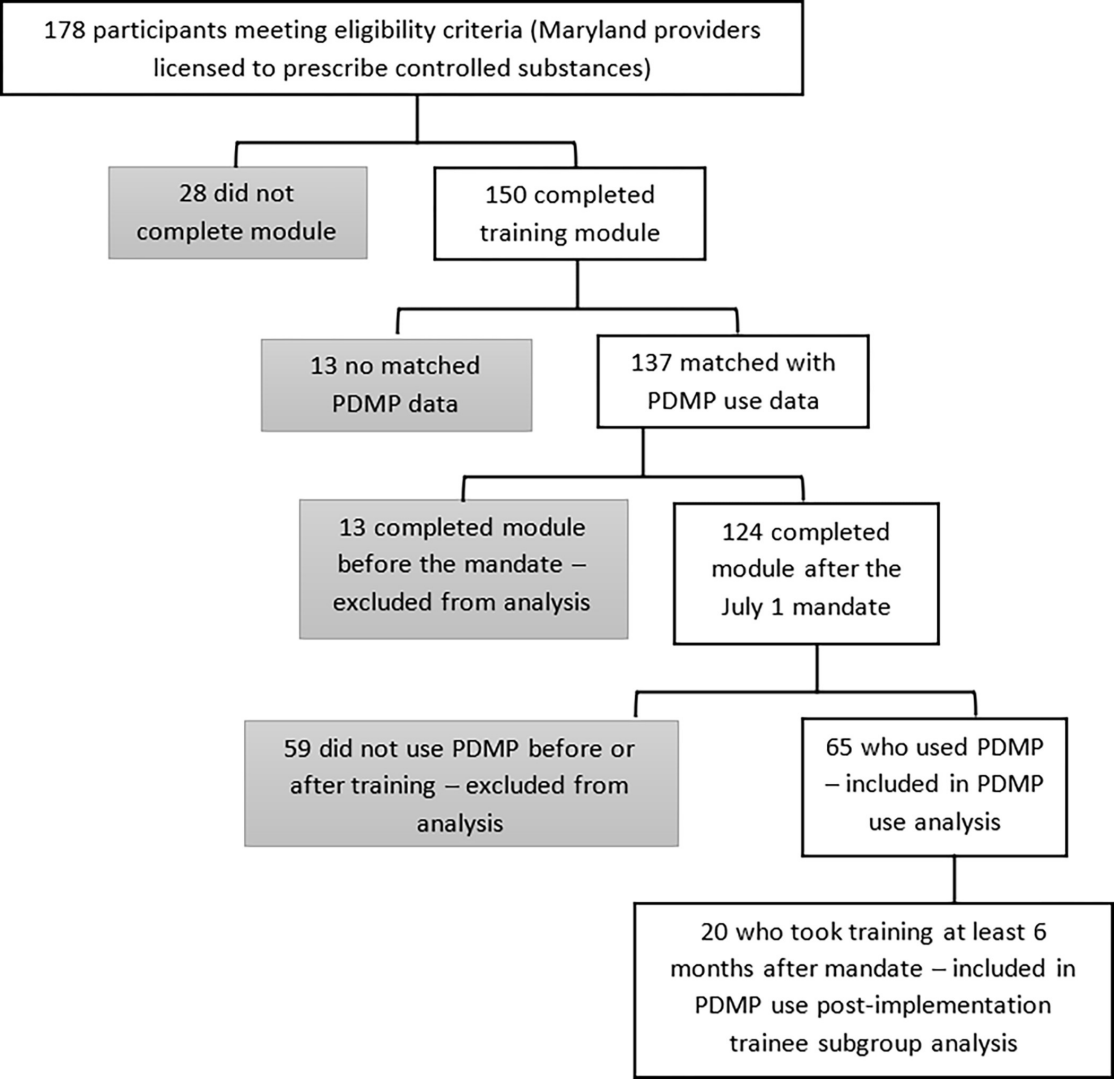
Participant data included in PDMP use analysis.

Of the 178 eligible participants who started the module, 150 (87%) completed the training module. We retrieved the real-world PDMP use data for 137 (91%) of these 150 participants for the period of 6-months pre-training and up to 6-months post-training (Fig 3). We excluded the 13 participants whose PDMP use data we could not match from the analysis of changes in using the PDMP following the training.

One hundred forty-five (97%) were physicians (defined as physicians, dentists, or podiatrists); four were nurse practitioners and one was a physician assistant. Most of the participants (57%) reported that they had been in practice for over 20 years; 34% of these identified as frequent opioid prescribers. Twenty-one (14%) participants reported they had been in practice for up to five years; 15 (10%) reported they had been in practice from 6–10 years and 28 (19%) reported 11–20 years of medical practice. We observed no significant difference in the years of experience between participants in our study population and Maryland licensed physicians, using the date of first medical registration as a surrogate for date entering practice.

Of the 150 participants, 24 reported they had not yet registered to use the PDMP at baseline; of these, eight reported their application was pending approval. However, we retrieved PDMP use data for 137 (91%) of the 150 participants, suggesting that some participants did not recall or realize that they had registered. The proportion of our study population with PDMP registration was comparable to that of the overall Maryland physician population (91% vs 86.6%, *p* = 0.37) [45]. We found no significant difference between frequent and infrequent opioid prescribers based on their PDMP registration status (90% vs. 80%; p<0.17). However, frequent prescribers were more likely than infrequent prescribers to report having ever used the PDMP (88% vs. 53%; p<0.05). Also, frequent opioid prescribers were more likely to report checking the PDMP frequently (73% vs. 29%; p<0.05). WC providers were more likely to have ever used PDMP than those who did not provide WC services in the previous year (77% vs. 49%; p<0.05). The remaining characteristics of the participants are summarized in Table 1. We presented five different clinical situations and measured agreement with importance of checking the PDMP in those situations. We then asked about PDMP behavior in the same clinical situations. Less than 50% of the study population reported checking the PDMP in all the clinical circumstances that they agreed they should.

**Table 1:** Participant demographic characteristics, attitudes and self-reported PDMP behavior at baseline.

	All Participants completing module^a^	Subgroup		*p*-value^d^
		Frequent prescribers^b^	Infrequent prescribers^c^	
	n = 150	n = 53	n = 97	
	n (%)	n (%)	n (%)	
Provided WC care in the past year	84 (56.0)	39 (73.6)	45 (46.4)	**0.002** *
Has PDMP registration	126 (84.0)	48 (90.6)	78 (80.4)	0.161
Of those registered, ever used the PDMP ^e^	83 of 126 (65.8)	42 of 48 (87.5)	41 of 78 (52.6)	**<0.001** *
Of those who ever used PDMP, reported frequent use ^f^	43 of 83 (51.8)	31 of 42 (73.8)	12 of 41 (29.3)	**<0.001** *
Situations where they consider PDMP to be important ^g^				
Suspected abuse	136 (90.7)	50 (94.3)	86 (88.7)	0.380
New patients	135 (90.0)	49 (92.5)	86 (88.7)	0.576
Pain managed elsewhere	135 (90.0)	50 (94.3)	85 (87.6)	0.259
New opioid treatment	128 (85.3)	47 (88.7)	81 (83.5)	0.474
Continue opioid treatment	128 (85.3)	44 (83.0)	84 (86.6)	0.631
Situations where they routinely check PDMP ^h^				
Suspected abuse	67 (44.7)	34 (64.2)	33 (34)	**<0.001** *
New patients	40 (26.7)	20 (37.7)	20 (20.6)	**0.038** *
Pain managed elsewhere	59 (39.3)	26 (49.1)	33 (34.0)	0.082
New opioid treatment	52 (34.7)	25 (47.2)	27 (27.8)	**0.028** *
Continue opioid treatment	47 (31.3)	25 (47.2)	22 (22.7)	**0.004** *

Abbreviations: WC, Worker’s compensation; PDMP, Prescription Drug Monitoring Program

^a^ The participants comprised 145 (96.7%) physicians, 4 (2.7%) nurse practitioners, and 1 (0.6%) physician assistant. The denominator is the number that responded to the question.

^b^ Frequent prescriber, any participant that prescribes opioids several times a day (N = 10), several times a week (N = 30), or about once per week (N = 13).

^c^ Infrequent prescriber, any participant that prescribes opioids a few times per month (18), about once per month (N = 7), a few times a year or less (N = 72).

^d^ *p*-value calculated using Fisher’s exact test with continuity correction.

^e^ Subset of “Has PDMP Registration”

^f^ Subset of “Has ever used PDMP”—frequency categories same as frequency of prescribing.

^g^ Agreement with the importance of the PDMP if in any of the subsequent options; Participants were asked in what circumstances they considered it important to query PDMP.

^h^ Reported routinely checking PDMP in patients if any of the subsequent options; Participants were asked in what circumstances they routinely queried PDMP in their practice.

* Alpha level of significance < 0.05

**Fig 4:**
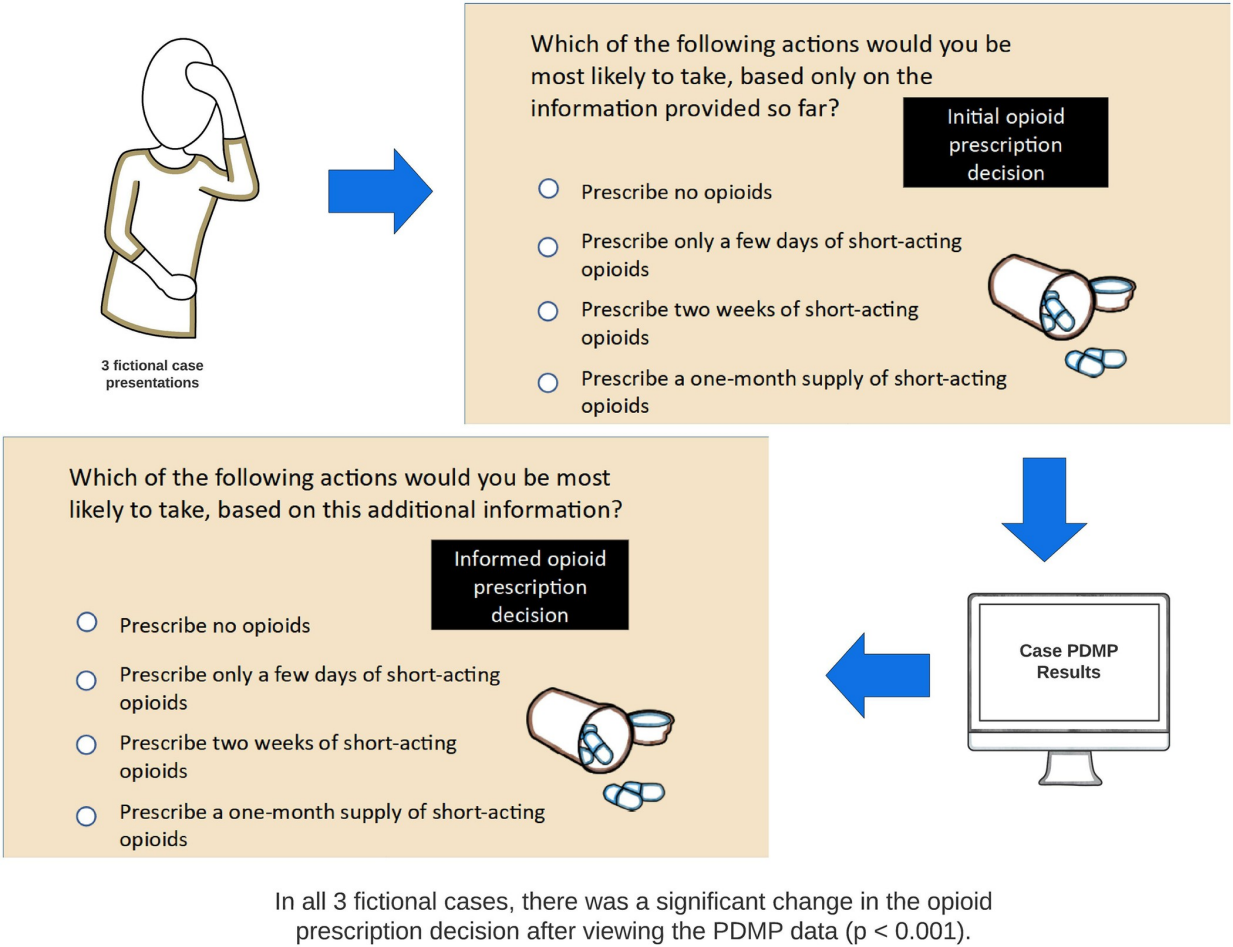
Training module case flow and changes in opioid prescription decisions based on PDMP data.

For each of the three fictional cases presented in the training module, participants’ opioid prescription treatment decisions changed significantly following the divulgence of the PDMP data (Fig 4 and S1 Table in S1 File).

We checked for differences between frequent and infrequent prescribers, and WC and non-WC providers, based on their opioid prescription decisions within the three specific fictional cases and found significant differences in both groups in case 1, a low back injury case. We found WC providers were also significantly more likely to revise their opioid prescription plan based on PDMP information in case 3, involving lost medication for a chronic pain condition (S2 Table in S1 File).

Based on the pre- and post-training surveys administered within the training module, there was a significant increase in the proportion of participants who considered it important to use the PDMP in most of the situations presented (Table 2 and S3 Table in S1 File). Post-training, 69% of all participants believed it was important to use the PDMP when seeing new patients (vs. 27% at baseline), and 126 (84%) indicated they would use the PDMP more frequently. Only four participants (2.7%) indicated that they did not routinely check the PDMP at baseline, and two (1.3%) indicated they did not plan to check the PDMP after the training. Nineteen participants (12.7%) reported checking the PDMP for current patients at least once at baseline, and 80 (53.3%) said they planned to do so at the end of training (p<0.001). Other baseline versus immediate-post-training self-reported changes are shown in Table 2.

**Table 2:** Self-reported situation-specific PDMP use at start of training and at end of training in all participants completing the module.

	At start of training (baseline)	At end of training	*p*-value^a^
	n = 150	n = 150	
	n (%)	n (%)	
***Clinical situation***	*In which scenarios do you routinely check the PDMP?*	*In which scenarios do you expect to routinely check the PDMP?*	
Suspected abuse	67 (44.7)	135 (90.0)	**<0.001** *
New patients	40 (26.7)	103 (68.7)	**<0.001** *
Pain managed elsewhere	47 (31.3)	118 (78.7)	**<0.001** *
New opioid treatment	59 (39.3)	138 (92.0)	**<0.001** *
Continue opioid treatment	52 (34.7)	128 (85.3)	**<0.001** *

Abbreviations: PDMP, Prescription Drug Monitoring Program

^a^ *p*-value calculated using McNemar’s test with continuity correction.

^b^ Post-training response option was “I do not plan to use the PDMP unless mandated.”

* Alpha level of significance < 0.05

Participants who did not access the PDMP at all in the six months before or after the training, based on PDMP access data, nevertheless showed a significant change in the intention to use the PDMP at the end of the module (S3 Table in S1 File).

Fifty-three (64%) of the 83 participants who rated the training module in the post-completion survey described it as excellent, 27 (33%) as good, and three (4%) as fair.

### Three-month follow-up

In the follow-up survey three months after completion of training data collection, we received responses from 32 of the 59 participants who agreed to follow-up contact by providing their email in the module. Fifteen reported using the PDMP about as much as they had planned to, 12 reported using it less than they had planned, and five reported using it more than they had anticipated. Of the 12 who reported using it less than expected, three indicated that they were prescribing opioids less.

### Actual use

We obtained actual use data on 137 of the 150 participants who completed the training. Of the 137, 13 completed the training early–before the PDMP mandate went into effect on July 1, 2018. Of the remaining 124, 30 (24%; 22 WC providers and 17 frequent opioid prescribers) participants used the PDMP frequently, while 59 (47.5%) participants did not use the PDMP in the six months before or the six months after the training. (Note that PDMP access data does not include potential access to PDMP data within organizational electronic health records.)

We ran a Wilcoxon signed-rank test on the 65 participants who took the training ***and*** used the PDMP after the mandate, for paired comparisons of use patterns. We excluded from this analysis the 13 (9.5%) participants who completed the training before the July 1, 2018 mandate and the 59 (47.5%) who never accessed the PDMP before or after the training. Of the 59 participants who never accessed the PDMP throughout the study period, 14 (24%) were self-reported frequent opioid prescribers, 28 (48%) were WC providers and 21 (36%) had been in practice for more than 20 years. Twelve of the 13 (92%) participants who took the training before the mandate were WC providers, three (23%) had been in practice for more than 20 years, and nine (69%) were frequent opioid prescribers.

The median rate of PDMP use for the 65 participants included was five per month in the pre-intervention period and 10 per month in the six-month post-intervention period. The rate of PDMP use increased in 39 of the 65 participants in the post-intervention period. The pre-training versus post-training analyses showed a significant increase in the rate of PDMP use in the months following the completion of the module compared to the months before the module in infrequent opioid prescribers (median increase: two per month; *p* = 0.014). Participants in practice less than 20 years showed a median increase of four use-cases per month (*p* = 0.039). In a sub-group analysis on participants (N = 20), who took the training after the state-implemented mandate had been in place for at least 6 months, there was a significant increase in the rate of PDMP use following the training (median increase:11/month; *p* = 0.003). However, the state mandate did not have a significant effect on the PDMP use in this sub-group of participants. The remaining results showing the difference in the rate of PDMP use pre- versus post-training are presented in Table 3.

**Table 3:** The difference in rate of PDMP use. Number of times PDMP was accessed per month before and after training based on participant self-reported characteristics.

	Participants^a^	Pre-training rate of PDMP use^b^	Post-training rate of PDMP use^c^	Difference in PDMP use	*p*-value^d^
	n (%)	Median (IQR)	Median (IQR)	Median (IQR)	
**All participants** ^**a**^	65 (100)	4.8 (0–22.5)	10 (2.0–32.0)	1 (-5.5 to 11.0)	0.352
Frequent opioid prescribers^e^	22 (33.8)	23 (6.0–181.5)	17.5 (3.3–74.5)	-3.5 (-45.8 to 3.0)	0.182
Infrequent opioid prescribers	43 (66.2)	2 (0–8.5)	7 (1.0–21.5)	2 (-1.1 to 11.0)	**0.014** *
WC providers	34 (52.3)	15 (0–58.5)	16 (6.5–60.3)	2.5 (-7.8 to 13.8)	0.494
Non-WC providers	31 (47.7)	2 (0–6.8)	3 (1.0–16.5)	1 (-4.2 to 4.5)	0.754
> 20 years in practice	35 (53.8)	7.5 (1.4–49.5)	10.0 (1.00–26.5)	-1 (-6 to 3.5)	0.491
< 20 years in practice	30 (46.2)	1.7 (0–11.0)	10.5 (3.3–35.0)	4 (1.0–23.0)	**0.039** *

Abbreviations: PDMP, Prescription Drug Monitoring Program; WC, workers’ compensation; IQR, Interquartile Range

^a^ Consists of all participants who took the training and used the PDMP after the July 1, 2018, PDMP mandate. 13 participants who took the training before the July 1 mandate, and 59 people who didn’t utilize the database at all for the period of observation before and 6 months period after taking the training were excluded from the analyses.

^b^ The rate of use is the number of times the PDMP database was accessed during a given period of observation. The pre-training period was the period from July 1, 2018, to the date when a participant completed the training.

^c^ The rate of use is the number of times the PDMP database was accessed during a given period of observation. The post-training period was 6 months following training completion for all groups of participants.

^d^ *p-*value calculated using Wilcoxon signed-rank test

^e^ Frequent prescribers are those who reported prescribing opioids several times a day, several times a week, or about once per week.

* Alpha level of significance < 0.05

Bivariate regression analyses showed a significant increase in the rate of PDMP usage among infrequent prescribers (OR:3.3, *p* = 0.027), and in participants who have been in practice for less than 20 years (OR: 3.9, *p* = 0.013) but no significant training impact was found on WC providers. This finding was confirmed in multi-model regression analysis with backward elimination in infrequent providers (OR:3.7, *p* = 0.041) and in participants in practice for less than 20 years (OR:9.4, *p* = 0.003). However, a significant training effect was also seen in the multi-model regression analysis for those who provide WC care (OR:6.6, *p* = 0.020), suggesting that for these providers the effect may have been related to some other factor or factors (S4 Table in S1 File).

## Discussion

This study evaluated the effectiveness of a brief online educational intervention in increasing use of the Maryland PDMP. Overall, the largest impact of the training appears to be on infrequent opioid prescribers and providers with less than 20 years of practice. These findings are consistent with those in a recent study on the impact of academic detailing about the PDMP on primary care provider use patterns, with the largest effect on late adopters (those who had not yet registered or used the PDMP months after this was mandated) [33]. Delcher et al. found in their analysis of oxycodone related overdoses in Florida that an increase of one PDMP query per provider per month resulted in a decline of oxycodone-caused deaths by 0.229 persons per month [47]. Our study found an increase in PDMP use by a median of two queries per month in the months following the training among infrequent opioid prescribers (*p* = 0.014) and four queries per month among those in practice less than 20 years (*p* ***=*** 0.039). This suggests that such training can be effective in increasing familiarity and comfort with the PDMP in those who are less familiar with it.

The finding of training benefit observed in the sub-group of participants who enrolled at least six months after the state PDMP mandate had been implemented suggests that the mandate itself may not be sufficient to influence decisions to use the PDMP. Although the conclusions are limited by the small sample size, this finding is consistent with research on the effect of mandates, which showed variations among states that were thought to relate to differences among state laws, including exceptions to the mandate requirements [48].

From the baseline questionnaire, we learned that WC care providers and frequent opioid prescribers reported more frequent PDMP use at baseline, and that there was a large overlap between these categories, with 74% of the frequent opioid prescribers also reporting providing WC services. We expected that those who prescribe opioids more frequently would have more occasion to use the PDMP, a finding noted by Lin et al. in their 2015 Maryland survey of Maryland physicians about the PDMP [27]. At baseline, there was strong agreement in the value of checking the PDMP in all five types of clinical situations presented with no difference at baseline among our participant groups. An important finding is a universal discrepancy between positive baseline PDMP attitudes and self-reported baseline PDMP behavior in study participants, with a large gap between belief in the value of the PDMP and self-reported use patterns, which is consistent with the findings of Lin et al. [27]. This suggests that educational efforts directed toward PDMP attitudes may not be useful.

Researchers in the design of medical curricula have suggested that education should address three domains [49–51]:

- Qualification refers to giving students the knowledge, skills, and understanding needed to “qualify” as competent practitioners.
- Socialization refers to practice norms, values, expectations, and specialty or cultural context.
- Subjectification refers to the aim for learners to end up as subjects, able to draw their own conclusions and make their own decisions, and relates to the key educational ideas of agency, autonomy, and responsibility.

The educational module in this study was designed to demonstrate to the learner the value of PDMP information in clinical case scenarios. We presented three realistic fictional cases about painful work-related injuries and asked study participants to make hypothetical clinical decisions. We then provided participants additional information from the PDMP in each case and asked each participant how this new information impacted their decision. There were no specific right or wrong answers in this module. Using this approach, we found statistically significant differences in the clinical decisions that participants made about opioid prescribing in each case before and after reviewing the PDMP information. By inviting participants to adjust their own clinical decisions based on data provided, rather than providing right or wrong answers, our educational approach aligns with the medical education domain of “subjectification” described by Biesta, which is often neglected in online medical education that focuses on the acquisition of knowledge [49–51].

In the fictional cases, both frequent prescribers and WC provider participants were more likely to modify their opioid treatment decisions after seeing PDMP data in the case involving acute low back pain, the bread-and-butter injury of WC care (S2 Table in S1 File). Given the high risk of poor outcomes in patients given inappropriate opioids for acute musculoskeletal injuries in WC care, this finding supports using a similar educational strategy focused on preventing early opioid prescriptions when not clinically indicated, targeted toward WC providers.

### Strengths

It is notoriously difficult to measure the actual impact of education on practice behaviors. We designed this study to also look at patterns of accessing the PDMP by participants before and after taking the training module, to measure any actual changes in PDMP use, one of the strengths of this study. This helped us overcome the well-documented propensity to overestimate compliance with best practices in self-reported behavior [52–56]. Participants acting as their own controls in the analyses involving comparisons before and after the module and in the case decision-making, is also a strength of the study design.

### Limitations

Our PDMP data did not capture institutional use (where PDMP data is presented when accessing organizational electronic health records), and we did not capture which participants were able to see PDMP data this way, so it is possible that our PDMP use data missed these participants accessing the PDMP, and therefore missed any changes in their use patterns. There was no attempt to randomly sample participants, therefore self-selected participants may not be representative of Maryland prescribing providers. Medical decisions made in fictional cases may not accurately reflect decisions made in real cases and we did not have access to data on opioid prescribing before or after the training. Because our initial plan to recruit WC care providers (a population of particular interest) from an insurance contact file was unsuccessful, we modified our protocol to query about WC practice, so that we could analyze this variable. The single question about providing WC care in the last year may not have effectively categorized WC care providers. We did not receive enough follow-up survey responses for meaningful comparisons in self-reported use after the training.

We adjusted for the potential interaction of the mandate instituted by the State of Maryland on July 1, 2018 by excluding participants who took the training before the mandate from the analyses and re-computed the analyses on a subgroup of participants who took the training at least 6 months following the mandate. We also excluded from the final analyses, subjects who never used PDMP any time 6 months before the training or in the 6 months after the training. Although we did not identify any difference between the excluded participants and the remaining study population, we cannot be certain whether these participants differ in any important way from those included in the analyses. This exclusion potentially limits the external validity of our study.

## Conclusion

This study demonstrated the effectiveness of a brief, engaging, online educational module on PDMP use by participants who were less familiar with the PDMP. This innovative “gradual reveal” method engages learners as their own teachers, incorporating additional information from the PDMP and revising their planned treatment in a way that promotes adherence to treatment guidelines that improve patient safety and clinical outcomes. Given that even modest increases in PDMP queries are associated with decreases in prescription opioid overdose deaths, the ability to shift the practice patterns of large numbers of prescribing providers using inexpensive online technology would have significant public health implications. This approach should be considered in future education aimed at decreasing early opioid prescribing in acute WC care, and for future educational strategies aimed at new PDMP registrants or physicians at an earlier phase in their careers. Different strategies may be needed for physicians who have been in practice for more than 20 years or who have already established specific practice patterns in using the PDMP. Online delivery of brief education demonstrating the value of PDMP data in clinical cases would be a cost-effective alternative to the more labor-intensive approach of academic detailing, which requires close to an hour of trainer time for each provider trained. Future research should address the feasibility, cost-effectiveness and impact of reaching larger groups of providers using online PDMP training, as well as methods for reaching providers who have more familiarity with the PDMP.

## Supporting information

S1 File Instruments used in this study, including module link, quiz questions and questionnaires. Additional results tables.
(DOCX)
