# Supplementary material for: Impact evaluation of a brief online training module on physician use of the Maryland, USA, Prescription Drug Monitoring Program
Source: PLoS One. 2022 Aug 9;17(8):e0272217. doi: 10.1371/journal.pone.0272217 (PMC9362906; doi:10.1371/journal.pone.0272217)
Supplement: S1 File — Additional results tables. (DOCX) [file pone.0272217.s001.docx]

Supplemental Materials

Contents

[Baseline questionnaire, at start of module 2](#_Toc96332045)

[The Module 5](#_Toc96332046)

[Interactive Case Questions used for the three fictional cases in the training module 6](#_Toc96332047)

[Post-module survey and evaluation embedded at the end of the training module 9](#_Toc96332048)

[Follow-up survey (select participants) sent to participants who agreed to contact by sharing email address in the module 11](#_Toc96332049)

[Supplemental Tables 12](#_Toc96332050)

[Table S1. Comparison of opioid prescription decisions in fictional cases before and after seeing PDMP data 12](#_Toc96332051)

[Table S2. Change in treatment decision in fictional cases based on opioid prescribing frequency and provision of WC care 13](#_Toc96332052)

[Table S3. Self-reported situation-specific PDMP use at start of training and immediately after training in a subset of participants who did not access the PDMP between December 1, 2017, and June 30, 2020. 14](#_Toc96332053)

[Table S4. Unadjusted and adjusted odds ratios from logistic regression analysis for participant factors associated with increased PDMP use following training. 15](#_Toc96332054)

# Baseline questionnaire, at start of module

Initial Survey on Use of the Prescription Drug Monitoring Program (PDMP)

Purpose

The purpose of this brief survey is to learn a little more about you, your practice, your attitudes and current behaviors related to the Maryland Prescription Drug Monitoring Program (PDMP). The information you provide will be separated from your identifying information for the purpose of analysis. We plan to compare the information about your attitudes and current behaviors at baseline with your attitudes immediately after you complete this module.

1. Which of the following describes your profession?

- Physician (includes Dentist, Podiatrist)
- Nurse Practitioner
- Physician Assistant

1. Have you treated any workers’ compensation claimants in the previous year?

- Yes
- No

1. Please fill in your Maryland Controlled Dangerous Substance (CDS) permit number here. This will help us match information in our project. You can look up your number here if you don't have it handy: <https://health.maryland.gov/cdssearch/SitePages/Home.aspx> . At this link, type in your name to retrieve your number. (Skip to the next question if you do not have a CDS permit.)

My CDS #: _____________

1. How many years have you been in practice?

- 0 to 5
- 6 to 10
- 11 to 20
- Over 20 years

1. How often do you prescribe opioids?

- Several times a day
- Several times per week
- About once a week
- A few times per month
- About once a month
- A few times per year or less

| For each statement below, please indicate your agreement or disagreement about the usefulness of the state Prescription Drug Monitoring Program (PDMP) for each of the following situations, with 1 indicating STRONGLY DISAGREE and 5 indicating STRONGLY AGREE. | 1 | 2 | 3 | 4 | 5 |
| --- | --- | --- | --- | --- | --- |
| The PDMP is useful when evaluating a patient I suspect of abusing or misusing controlled substances. |  |  |  |  |  |
| The PDMP is useful for learning more about the past prescriptions written for patients new to my practice. |  |  |  |  |  |
| The PDMP is useful for researching the prescription history of a patient whose pain is managed by another provider. |  |  |  |  |  |
| The PDMP is useful for making a decision about whether to write a new prescription for a controlled substance. |  |  |  |  |  |
| The PDMP is useful for making a decision about continuing previous controlled substance prescriptions. |  |  |  |  |  |

1. Have you registered with the Chesapeake Regional Information System for our Patients (CRISP) to access the Maryland Prescription Drug Monitoring Program (PDMP)?

- Yes
- No
- My application is processing
- I don’t know

1. If you have NOT registered with CRISP to access PDMP data, why have you not done so? (Choose all that apply.)

- I was not aware of the PDMP or requirement to register
- I do not prescribe controlled substances
- I do not know how to register for PDMP data access
- I do not have enough time to register for PDMP data access
- I am not likely to use PDMP data in my practice
- Other (please specify)

1. If you selected other reason for not registering, please describe: ______________
2. If you answered that you HAVE access to the PDMP, have you ever used PDMP data in CRISP to examine the controlled substance use of your own patients?

- Yes
- No

1. How often do you check/query the PDMP?

- Several times a day
- Several times per week
- About once a week
- A few times per month
- About once a month
- A few times per year or less

1. In which scenarios do you routinely check the PDMP? (Check all that apply)

- For patients I suspect of aberrant drug behavior
- For new patients
- For patients to whom I prescribe a controlled substance for the first time
- For patients to whom I continue prescriptions for controlled substances
- For patients who are receiving controlled substances from other providers
- For current patients at least once
- I do not routinely check the PDMP

1. How often do you use the PDMP data in CRISP to examine controlled prescription drug utilization among patients *in whom you suspect* prescription drug abuse?

- Never
- 1-24% of the time
- 24-49% of the time
- 50-74% of the time
- 75-99% of the time
- Always

1. How often do you use the PDMP data in CRISP to examine controlled prescription drug utilization among patients *in whom you do not suspect* prescription drug abuse?

- Never
- 1-24% of the time
- 24-49% of the time
- 50-74% of the time
- 75-99% of the time
- Always

1. How often do you check the PDMP before prescribing opioids to a patient *for the first time*?

- Never
- 1-24% of the time
- 24-49% of the time
- 50-74% of the time
- 75-99% of the time
- Always

1. How often do you check the PDMP before *refilling* an opioid prescription?

- Never
- 1-24% of the time
- 24-49% of the time
- 50-74% of the time
- 75-99% of the time
- Always

1. In which scenarios do you expect to routinely check the PDMP? (Check all that apply)

- For patients I suspect of aberrant drug behavior
- For new patients
- For patients to whom I prescribe a controlled substance for the first time
- For patients to whom I continue prescriptions for controlled substances
- For patients who are receiving controlled substances from other providers
- For current patients at least once
- I do not expect to routinely check the PDMP

1. In your practice do you use or plan to use a delegate (other office staff registered to access PDMP) to check the PDMP?

- Yes
- No
- I’m not sure

The Module

The module minus the questionnaires can be accessed at this link: <https://ispri.ng/KWDXN> [
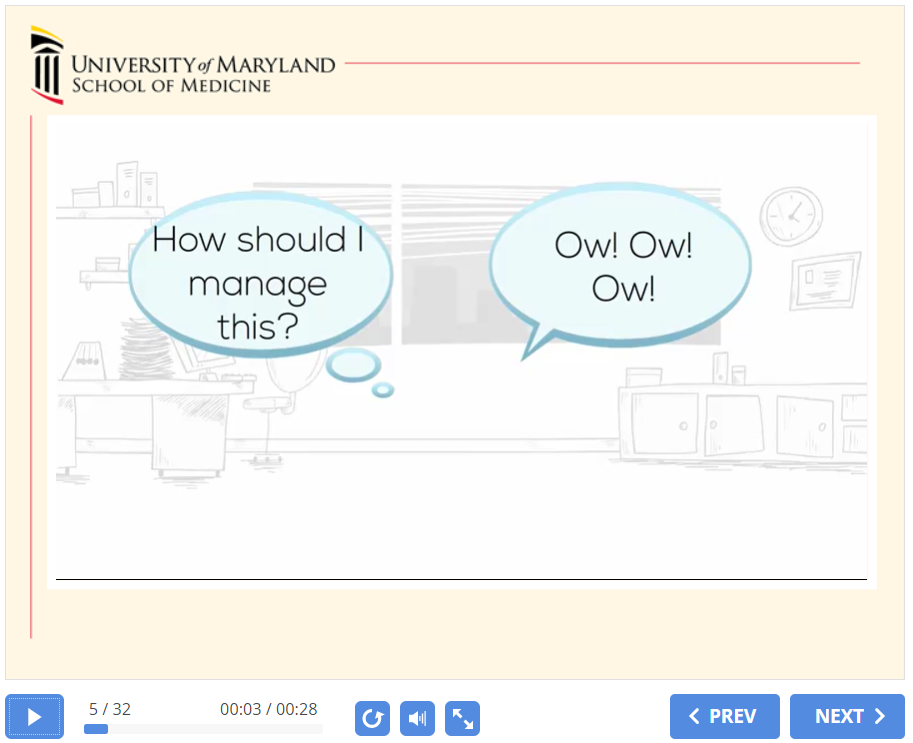
](https://ispri.ng/KWDXN)

# Interactive Case Questions used for the three fictional cases in the training module

**Case 1**

*Initial Plan for Case 1*

Which of the following actions would you be most likely to take, based only on the information provided so far?

- Prescribe no opioids
- Prescribe only a few days of short-acting opioids
- Prescribe two weeks of short-acting opioids
- Prescribe a one-month supply of short-acting opioids

Which of the following additional actions would you take in managing this patient? (Check all that apply.)

- Refer to physical therapy
- Contact his primary care doctor
- Prescribe non-opioid medications for pain
- Contact workers' compensation case manager
- Obtain urine for toxicology testing
- Check the PDMP

*Informed Plan for Case 1*

Which of the following actions would you be most likely to take, based on this additional information?

- Prescribe no opioids
- Prescribe only a few days of short-acting opioids
- Prescribe two weeks of short-acting opioids
- Prescribe a one-month supply of short-acting opioids

Which of the following additional actions may be appropriate to consider in this case? (Check all that apply.)

- Contact the workers' compensation case manager
- Open a conversation with the patient about his prescription history
- Obtain urine for toxicology testing
- Contact his primary care doctor
- Prescribe non-opioid medication for pain
- Refer to physical therapy

Did your management plan change with the PDMP information?

- Yes
- No

**Case 2**

*Initial Plan for Case 2*

Which of the following actions would you be most likely to take, based only on the information provided so far?

- Prescribe no opioids
- Prescribe only a few days of short-acting opioids
- Prescribe two weeks of short-acting opioids
- Prescribe a one-month supply of short-acting opioids

Which additional actions would you consider in this case? (Check all that apply.)

- Contact the primary care provider
- Prescribe non-opioid medication for pain
- Refer to physical therapy
- Obtain urine for toxicology testing
- Contact the workers' compensation case manager
- Check the PDMP
- Refer to behavioral therapy

*Informed Plan for Case 2*

Which of the following actions would you be most likely to take, based on this additional information?

- Prescribe no opioids
- Prescribe only a few days of short-acting opioids
- Prescribe two weeks of short-acting opioids
- Prescribe a one-month supply of short-acting opioids

Which of the following additional actions may be appropriate to consider in this case?

- Contact the primary care provider
- Prescribe non-opioid medication for pain
- Refer to physical therapy
- Obtain urine for toxicology testing
- Contact the workers' compensation case manager
- Refer to behavioral therapy
- Offer non-pharmacologic treatment
- Discuss your concerns about drug interactions and adverse effects with the patient

Did your management plan change with the PDMP information?

- Yes
- No

**Case 3**

*Initial Plan for Case 3*

Which of the following actions would you be most likely to take, based only on the information provided so far?

- Prescribe no opioids
- Prescribe only a few days of short-acting opioids
- Prescribe two weeks of his usual medication regimen
- Prescribe a few days of his usual medication regimen

Which additional actions would you consider taking at this point? (Check all that apply.)

- Check the PDMP
- Obtain urine for toxicology testing
- Contact the police
- Contact the treating neurologist
- Contact the workers' compensation case manager
- Prescribe non-opioid medication for pain
- Evaluate case for adequate past trial of non-pharmacologic treatment options

*Informed Plan for Case 3*

Which of the following actions would you be most likely to take, based on the additional information?

- Prescribe no opioids
- Prescribe only a few days of short-acting opioids
- Prescribe two weeks of his usual medication regimen
- Prescribe a few days of his usual medication regimen

Which of the following additional actions may be appropriate to consider in this case? (Check all that apply.)

- Obtain urine for toxicology testing
- Contact the police
- Contact the treating neurologist
- Contact the workers' compensation case manager
- Prescribe non-opioid medication for pain
- Replace his non-opioid medications that were stolen
- Evaluate case for adequate past trial of non-pharmacologic treatment options

Did your management plan change with the PDMP information?

- Yes
- No

# Post-module survey and evaluation embedded at the end of the training module

Purpose

This survey is designed to try to capture any differences in attitudes related to using the state Prescription Drug Monitoring Program (PDMP), as well as to assess the educational value of the module.

We appreciate you taking the time to complete it.

After viewing this presentation, for which scenarios do you plan on checking the PDMP?

- For patients I suspect of aberrant drug behavior
- For new patients
- For patients to whom I prescribe a controlled substance for the first time
- For patients to whom I continue prescriptions for controlled substances
- For patients who are receiving controlled substances from other providers
- For current patients at least once
- I do not plan to use the PDMP unless mandated

For each statement below, please indicate your agreement or disagreement about the usefulness of the state Prescription Drug Monitoring Program (PDMP) for each of the following situations, with 1 indicating STRONGLY DISAGREE and 5 indicating STRONGLY AGREE.

|  | 1 | 2 | 3 | 4 | 5 |
| --- | --- | --- | --- | --- | --- |
| The PDMP is useful when evaluating a patient I suspect of abusing or misusing controlled substances. |  |  |  |  |  |
| The PDMP is useful for learning more about the past prescriptions written for patients new to my practice. |  |  |  |  |  |
| The PDMP is useful for researching the prescription history of a patient whose pain is managed by another provider. |  |  |  |  |  |
| The PDMP is useful for making a decision about whether to write a new prescription for a controlled substance. |  |  |  |  |  |
| The PDMP is useful for making a decision about continuing previous controlled substance prescriptions. |  |  |  |  |  |

After viewing this presentation, do you plan to access the PDMP more frequently?

- Yes
- No

How would you rate the overall quality of this educational activity?

- Poor
- Fair
- Good
- Excellent

For each statement below, please indicate your agreement or disagreement about the effectiveness of this educational activity with 1 indicating STRONGLY DISAGREE and 5 indicating STRONGLY AGREE.

|  | 1 | 2 | 3 | 4 | 5 |
| --- | --- | --- | --- | --- | --- |
| This activity increased my ability to identify cases for which PDMP consultation is appropriate. |  |  |  |  |  |
| This activity increased my understanding of how to incorporate PDMP information into clinical decision-making. |  |  |  |  |  |
| This activity helped me recognize the value of the PDMP in supporting safe prescribing of opioids. |  |  |  |  |  |

Please describe any changes you plan to make as a result of what you learned in this activity:

__________________________________________________________________________________________________________________________________________________________________________

Did you feel this activity contained any commercially biased information?

- Yes
- No

If you answered “Yes” to the previous question, please explain: __________________________________________________________________________________________________________________________________________________________________________

May we contact you in the next several months to participate in a brief follow-up survey? If yes, please type your e-mail below: __________________

We welcome any feedback on this module or experience here:

__________________________________________________________________________________________________________________________________________________________________________

Thank you!

Thank you for taking the time to complete this survey and participate in this project. You may claim up to 30 minutes of free continuing medical education (CME) credit, following instructions at the Center for Innovative Pharmacy Solutions (CIPS) portal. We will send all participants who completed this module and provided an e-mail address a summary of our findings when we have completed our analysis. We appreciate you taking the time to assist us!

# Follow-up survey (select participants) sent to participants who agreed to contact by sharing email address in the module

For the follow-up survey to selected participants who consented to follow-up by providing their e-mail address in the post-training evaluation, we plan to use selected questions from the initial questionnaires, with the following additions.

Intro: The following questions relate to the Prescription Drug Monitoring Program training module you took several months ago at the University of Maryland Center for Innovative Pharmacy Solutions website. This survey addresses your experiences since that time. It should take no more than 10 minutes to complete.

Which of the following most closely reflects your experience with the PDMP since taking the module?

- I have used the PDMP as much as I planned to
- I have used the PDMP more than I planned to
- I have used the PDMP less than I planned to

If you used the PDMP less than you planned to, please indicate which, if any, of the following factors contributed. Please choose all that apply.

- I made the decision to prescribe opioids less frequently than I thought I would.
- Checking the PDMP did not seem necessary in as many cases in which I prescribed opioids, than I anticipated.
- I found the PDMP too difficult to use.
- I found checking the PDMP too time-consuming.
- Other: __________________________________

# Supplemental Tables

## Table S1. Comparison of opioid prescription decisions in fictional cases before and after seeing PDMP data

|  | Did the treatment plan include prescribing any opioids *AFTER* PDMP data disclosure? | | | |
| --- | --- | --- | --- | --- |
|  | **Yes** | **No** | **Total** | ***p*-value^a^** |
| Did the treatment plan include prescribing any opioids *BEFORE* PDMP data disclosure? |  | | | |
| Case 1^b^ |  | | | |
| Yes | 3 | 58 | 61 | **<0.001*** |
| No | 0 | 89 | 89 |  |
| Total | 3 | 147 | 150 |  |
| Case 2 ^c^ |  | | | |
| Yes | 23 | 33 | 56 | **<0.001*** |
| No | 6 | 88 | 94 |  |
| Total | 29 | 121 | 150 |  |
| Case 3 ^d^ |  | | | |
| Yes | 42 | 6 | 48 | **<0.001*** |
| No | 58 | 44 | 102 |  |
| Total | 100 | 50 | 150 |  |

Abbreviations: PDMP, Prescription Drug Monitoring Program

^a^ *p*-value calculated using McNemar’s test with continuity correction

^b^ Case 1 presented a patient with a history of an acute low back injury at work; PDMP data indicated multiple recent opioid prescriptions from different sources.

^c^ Case 2 presented a patient with head, neck and shoulder pain following a motor vehicle accident a few days before; PDMP data indicated chronic use of multiple controlled substances with high risk for sedation and dangerous interactions.

^d^ Case 3 presented a patient with a history of complex regional pain syndrome, whose long-acting opioid medication was stolen on a business trip; PDMP data supported the patient’s prescription history.

* Alpha level of significance < 0.05

## Table S2. Change in treatment decision in fictional cases based on opioid prescribing frequency and provision of WC care

|  | **Frequent Prescribers** | **Infrequent Prescribers** | ***p*-value^a^** | **WC Care Provided** | **No WC Care Provided** | ***p*-value^b^** |
| --- | --- | --- | --- | --- | --- | --- |
|  | **n (%)** | **n (%)** |  | **n (%)** | **n (%)** |  |
| **Subgroup total** | 53 (35.3) | 97 (64.7) |  | 84 (56.0) | 66 (44.0) |  |
| **Case 1 *initial* decision included any opioids** | 29 (54.7) | 32 (33.0) | **0.015*** | 39 (46.4) | 22 (33.3) | 0.132 |
| **Case 1 *informed* decision included any opioids** | 1 (1.9) | 2 (2.1) | 1 | 1 (1.2) | 2 (3.0) | 0.583 |
| **Case 1 treatment decisions changed with PDMP information** | 49 (92.5) | 71 (73.2) | **0.005*** | 74 (88.1) | 46 (69.7) | **0.007*** |
| **Case 2 *initial* decision included any opioids** | 21 (39.6) | 35 (36.1) | 0.725 | 30 (35.7) | 26 (39.4) | 0.734 |
| **Case 2 *informed* decision included any opioids** | 13 (24.5) | 16 (16.5) | 0.281 | 17 (20.2) | 12 (18.2) | 0.836 |
| **Case 2 treatment decisions changed with PDMP information** | 40 (75.4) | 70 (72.2) | 0.704 | 62 (73.8) | 48 (72.7) | 1 |
| **Case 3 *initial* decision included any opioids** | 20 (37.7) | 28 (28.9) | 0.278 | 29 (34.5) | 19 (28.8) | 0.485 |
| **Case 3 *informed* decision included any opioids** | 41 (77.3) | 59 (60.8) | **0.047*** | 48 (57.1) | 52 (78.8) | **0.006*** |
| **Case 3 treatment decisions changed with PDMP information** | 49 (92.5) | 80 (82.5) | 0.138 | 68 (80.9) | 61 (92.4) | 0.058 |

Abbreviations: WC, Worker’s compensation; PDMP, Prescription Drug Monitoring Program

^a,b^ *p*-values calculated using Fischer’s exact test with continuity correction.

* Alpha level of significance < 0.05

## Table S3. Self-reported situation-specific PDMP use at start of training and immediately after training in a subset of participants who did not access the PDMP between December 1, 2017, and June 30, 2020.

|  | **At start of training (baseline)** | **At end of training** | ***p*-value^a^** |
| --- | --- | --- | --- |
|  | **n = 59** | **n = 59** |  |
|  | **n (%)** | **n (%)** |  |
| ***Clinical situation*** | *In which scenarios do you routinely check the PDMP?* | *In which scenarios do you expect to routinely check the PDMP?* |  |
| **New patients** | 10 (16.9) | 40 (67.8) | **<0.001*** |
| **I do not routinely check the PDMP^b^** | 2 (3.4) | 1 (1.7) | 0.984 |
| **For current patients at least once** | 1 (1.7) | 28 (47.5) | **<0.001*** |
| **For patients to whom I prescribe a controlled substance for the first time** | 14 (23.7) | 55 (93.2) | **<0.001*** |
| **For patients to whom I continue prescriptions for controlled substances** | 14 (23.7) | 49 (83.1) | **<0.001*** |
| **For patients who are receiving controlled substances from other providers** | 9 (15.3) | 43 (1/7) | **<0.001*** |
| **For patients I suspect of aberrant drug behavior** | 15 (25.4) | 52 (88.1) | **<0.001*** |

Abbreviations: PDMP, Prescription Drug Monitoring Program

^a^ *p*-values were calculated using McNemar test with continuity correction.

^b^ Post-training response was “I do not plan to use the PDMP unless mandated”

* Alpha level of significance < 0.05

## Table S4. Unadjusted and adjusted odds ratios from logistic regression analysis for participant factors associated with increased PDMP use following training.

|  | ***Unadjusted estimate*** | | ***Adjusted estimate*** | |
| --- | --- | --- | --- | --- |
|  | **OR (95% CI)** | ***p*-value^a^** | **OR (95% CI)** | ***p*-value^a^** |
| **Infrequent opioid prescribers^b^** | 3.3 (1.2 - 10.0) | **0.027*** | 3.7 (1.0-15.0 | **0.041*** |
| **WC providers^c^** | 1.5 (0.6 - 4.2) | 0.42 | 6.6 (1.6 - 36.8) | **0.020*** |
| **Less than 20 years in practice^d^** | 3.9 (1.4 -12.1) | **0.013*** | 9.4 (2.4-51.4) | **0.003*** |
| **Participants who never used PDMP^e^** | 3.1 (0.8 - 15.0) | 0.120 | 3.9 (0.8 - 29.0) | 0.125 |
| **Infrequent PDMP users^f^** | 0.5 (0.2 – 1.5) | 0.235 | - | - |

Abbreviations: OR, Odds Ratio; WC, workers’ compensation; PDMP, Prescription Drug Monitoring Program; CI, Confidence Interval

^a^ Using the median rate of change in use (one use case per month) of the observed pairwise differences in all participants as the cut-off, we binarized the observed changes in the rate of PDMP use before and after training. We then conducted bivariate and multivariate logistics regression models to estimate the odds of observing a pairwise change equal to or greater than the median. We selected the best model for the adjusted OR using a stepwise logistic regression model (forward selection and backward elimination).

^b^ Infrequent opioid prescribers: Participants reporting prescribing opioids a few times per month, about once per month, a few times a year or less.

^c^ Participants reporting providing WC related care in the last year

^d^ Participants reporting less than 20 years in practice

^e^ Participants reporting never having used the PDMP

^f^ Infrequent PDMP users: Participants reporting checking the PDMP a few times per month, about once per month, a few times a year or less.

* Alpha level of significance < 0.05
